# Evident Exception in Clinical Practice Not Sufficient to Break Traditional Hypothesis

**DOI:** 10.1371/journal.pmed.0030120

**Published:** 2006-02-28

**Authors:** Milan Sarek

**Affiliations:** **1**Charles University in PraguePragueCzech Republic

I read with interest the Essay written by Jeffrey R. Lacasse and Jonathan Leo [ 1]. They have found that pharmaceutical companies marketing selective serotonin reuptake inhibitors (SSRIs) in the United States commonly declare that SSRIs correct chemical imbalance in depression caused by a lack of serotonin. The authors argue that serotonin deficiency in depression is scientifically unsupportable; therefore, statements about the lack of serotonin should not be used.

I agree with the authors. Nevertheless, I would like to add information about another drug that supports their arguments. I believe this drug should be mentioned in every article describing the serotonin system and depression. The drug is not authorized in English-speaking countries (see Micromedex Health Series at http://www.micromedex.com), and this may possibly explain why the information about its controversial mechanism is not largely shared in scientific literature.

The name of this drug is tianeptine. It has been used for over one decade in several European and some other countries (e.g., Brazil, India, Russian Federation, Thailand, Turkey, etc.) to relieve depression. Tianeptine increases serotonin reuptake; therefore, it has the opposite effect on the serotonin system compared with that of SSRIs (see Micromedex Health Series at http://www.micromedex.com) [ 2, 3]. The antidepressant efficacy of tianeptine versus tricyclics and SSRIs has been demonstrated in several studies [ 2, 3]. Because of this opposite action on the serotonin system, it has a different adverse event profile; e.g., the elevated frequency of sexual dysfunction commonly seen after SSRIs is not so frequently observed with tianeptine [ 4]. The mechanism of tianeptine's action is difficult to understand, but comparing this drug with other antidepressants provides us with an intellectual challenge [ 5].

Tianeptine has been in clinical use for over 12 years in the Czech Republic, but statements about serotonin deficiency in depression are still presented to the public by the media and in the patient information leaflets accompanying the majority of registered SSRIs. One of the reasons for this may be that it is difficult for the single producer of tianeptine to argue against the “traditional” hypothesis that supports the numerous leading pharmaceutical companies that market SSRIs.

For these reasons, I propose that the most direct way to conquer the unsupportable, but widely proclaimed, concept of serotonin deficiency in depression is to act through regulatory authorities who are responsible for the information given to the public and who also have the power to change the information that is provided about registered drugs.
